# Simulated Weightlessness Perturbs the Intestinal Metabolomic Profile of Rats

**DOI:** 10.3389/fphys.2019.01279

**Published:** 2019-10-15

**Authors:** Mingliang Jin, Jiaojiao Wang, Hao Zhang, Hongbin Zhou, Ke Zhao

**Affiliations:** ^1^College of Animal Sciences, Zhejiang University, Hangzhou, China; ^2^School of Life Sciences, Northwestern Polytechnical University, Xi’an, China; ^3^Dalian Chengsan Animal Husbandry Co., Ltd., Dalian, China; ^4^College of Food Engineering and Nutritional Science, Shaanxi Normal University, Xi’an, China

**Keywords:** simulated weightlessness, intestine, metabolomics, microbiota, immunity

## Abstract

Recently, disorders of intestinal homeostasis in the space environment have been extensively demonstrated. Accumulating evidence have suggested microgravity and simulated weightlessness could induce dysbiosis of intestinal microbiota, which may contribute to the bowel symptoms during spaceflight. However, the specific responses of intestinal metabolome under simulated weightlessness and its relationship with the intestinal microbiome and immune characteristics remain largely unknown. In the current study, 20 adult Sprague-Dawley (SD) rats were randomly divided into the control group and the simulated weightlessness group using a hindlimb unloading model. The metabolomic profiling of cecal contents from eight rats of each group was investigated by gas chromatography-time of flight/mass spectrometry. The significantly different metabolites, biomarkers, and related pathways were identified. Multivariate analysis, such as principal component analysis and orthogonal projections to latent structures-discriminant analysis, demonstrated an obvious separation between the control group and the simulated weightlessness group. Significantly different metabolites, such as xylose, sinapinic acid, indolelactate, and digalacturonic acid, were identified, which participate in mainly pyrimidine metabolism, pentose and glucuronate interconversions, and valine, leucine and isoleucine metabolism. Cytidine-5′-monophosphate, 4-hydroxypyridine, and phloretic acid were determined as pivotal biomarkers under simulated weightlessness. Moreover, the significantly different metabolites were remarkably correlated with dysbiosis of the intestinal microbiota and disturbance of immunological characteristics induced by simulated weightlessness. These metabolic features provide crucial candidates for therapeutic targets for metabolic disorders under weightlessness.

## Introduction

Recently, adaptive alternations of digestive physiology and their functional consequence in the space environment have been extensively demonstrated (Garrett-Bakelman et al., 2019). In particular, disorders of intestinal homeostasis, such as disruption of intestinal structure, decrease in nutritional digestion and absorption, dysfunction of intestinal immunity, and dysbiosis of the intestinal microbiota, were found to be induced by microgravity and simulated weightlessness (Rabot et al., 2000; Li et al., 2015; Ritchie et al., 2015; Shi et al., 2017; Jin et al., 2018; Wang et al., 2018). For instance, the NASA Twins Study suggested that long-duration spaceflight obviously changed the gastrointestinal microbiota and the related microbial metabolism of crewmembers during the 340-day mission onboard the International Space Station (Garrett-Bakelman et al., 2019). In addition, 7 days of simulated weightlessness might lead to the disruption of intestinal barrier function and thus the disturbance of normal defense and metabolic function of intestinal epithelial cells, based on a proteomic approach (Wang et al., 2018). Combined with the improved pathogenic features of the microbiome under weightlessness, such as increased virulence and biofilm formation, and enhanced resistance to antibiotics (Liu, 2017; Aunins et al., 2018; Sielaff et al., 2019), the intestinal tract is supposed to be much more susceptible to risks, such as inflammatory bowel disease, than other organ systems.

The microbiota is involved in the regulation of intestinal homeostasis through mainly the modulation of signaling pathways by microbe-derived metabolites (Jin et al., 2017). These small-molecule metabolites play a crucial role not only in the selection of microbiome but also in the establishment of the metabolic signaling network (Vernocchi et al., 2016). Thus, they serve as intermediaries between the host and the gut microbiota. Based on the development of “omics” technologies, metabolomics has become an unprecedented and powerful approach to unravel the essential and comprehensive alternations in diverse biological systems (Smirnov et al., 2016). Metabolomics has been used to elucidate the human urinary metabolic responses to simulated weightlessness using a 45-day 6° head-down tilt bed rest model (Chen et al., 2016).

In our previous study, intestinal barrier function responses of rats under simulated weightlessness were investigated using a well-established ground-based hindlimb unloading model, which is also known as the tail suspension model (Jin et al., 2018). This model can be used to mimic certain physiological effects, such as headward fluid shift, redistribution of the blood, and inadequate blood and oxygen supply to the gastrointestinal tract (Globus and Morey-Holton, 2016). We revealed disruption of intestinal barrier functions under simulated weightlessness, such as damaged structural features, dysbiosis of the microbiota, increased proinflammatory cytokine levels, and activation of related signaling pathways (Jin et al., 2018). Due to the crucial roles of microbial metabolites, insight into the responses of the intestinal metabolomic profile under simulated weightlessness and its relationship with the intestinal microbiome and immune characteristics is still needed, which will also provide evidence regarding the feasibility of the hindlimb unloading model for simulated weightlessness research. In the current study, intestinal metabolomic profiles under simulated weightlessness were investigated using hindlimb unloading and gas chromatography-time of flight/mass spectrometry (GC-TOF/MS). Furthermore, the relationship of significantly different metabolites with altered microbiome and immune characteristics was analyzed. The findings may provide deep insights into and detailed information on systematic responses, especially intestinal homeostasis, under simulated weightlessness.

## Materials and Methods

### Experimental Design

All animal experiments were approved by the Institutional Animal Care and Use Committee of Northwestern Polytechnical University and carried out in accordance with the institutional ethical guidelines of experimental animals. Twenty male adult Sprague-Dawley (SD) rats (199 ± 15.7 g), which were obtained from the Experimental Animal Center, Xi’an Jiaotong University, were randomly divided into two groups with 10 rats each: the control group (CON) and the hindlimb unloading group (SUS). Animals in the SUS group were tail-suspended at a 30° head-down tilt without load bearing on the hindlimbs, according to our previous report (Jin et al., 2018). All the rats were housed in plastic cages individually at room temperature (22 ± 1°C) under a 12 h light-dark cycle and provided with a commercial pellet diet and water *ad libitum*. The period of the animal experiment lasted for 21 days. At the end of the study, the rats were fasted for 12 h and anesthetized with ether.

### Concentrations of IL-4, IFN-γ, DAO, and ET in Serum and SIgA in the Ileum

The concentrations of interleukin-4 (IL-4), interferon- γ (IFN-γ), diamine oxidase (DAO), and endotoxin (ET) in serum and the level of secretory immunoglobulin A (SIgA) in the ileum were determined by ELISA assay, the related methods and results of which were reported in our previous study (Jin et al., 2018). Briefly, blood samples were collected and centrifuged at 1000 × *g* for 10 min at 4°C, and then the serum was separated. Ileums were immediately excised, homogenized in normal saline, and centrifuged at 10000 × *g* for 5 min at 4°C. The resulting supernatant fractions of homogenates were collected. A Synergy HT Multi-Detection Microplate Reader (Bio-Tek) was used with corresponding commercially available kits (BD Biosciences Pharmingen, San Diego, CA, United States).

### Characterization of the Intestinal Microbiota

Cecal contents collected were stored in freezing tubes at −80°C until further microbiome and metabolome analysis. Total bacterial DNA extraction from each cecal content sample, PCR amplification, and 16S rRNA gene sequencing were carried out according to the methods in our previous investigation (Jin et al., 2018). Briefly, bacterial DNA was extracted using an E.Z.N.A.^®^ Genomic DNA Isolation Kit (Omega Bio-Tek, Doraville, GA, United States). The V1–V3 hypervariable regions of the 16S rRNA gene were amplified by PCR with the broadly conserved primers 27F and 533R and sequenced using the Roche Genome Sequencer GS FLX Titanium platform (454 Life Sciences) at Shanghai Majorbio Bio-Pharm Technology Co., Ltd. (Shanghai, China). The 16S rRNA gene sequences were deposited in the NCBI Sequence Read Archive under BioProject PRJNA472839 with the accession number SRP148837.

Microbial community analysis was performed as described, the detailed results of which were reported in our previous report (Jin et al., 2018). Briefly, read processing and quality control (length and quality, chimera removal, primer trimming, and merging of pair-end reads) was performed using the QIIME pipeline. The resulting sequences were further analyzed using the following: (1) non-taxonomic-based clustering algorithms for operational taxonomic units (OTUs) by USEARCH with a 97% similarity cutoff; (2) a taxonomic-based approach from the phylum to genus levels using the Ribosomal Database Project (RDP) MultiClassifier tool; and (3) differentially abundant taxa identification using the linear discriminant analysis (LDA) effect size (LEfSe) method^1^.

### Metabolomic Profiling by GC-TOF/MS

Sample preparation, metabolite measurement by GC-TOF/MS, hierarchical clustering, biomarker analysis, and related pathway characterization were performed according to the method described in our previous report (Jin et al., 2019). Briefly, cecal contents from eight randomly selected rats of each group were extracted with methanol and chloroform (3:1) and derivatized using methoxy amination hydrochloride (20 mg/ml in pyridine) and derivatization regent (BSTFA + TMCS, 99:1). GC-TOF/MS was carried out using an Agilent 7890 gas chromatograph system (Agilent 7890A, Agilent Technologies, United States) coupled to a Pegasus HT time-of-flight mass spectrometer (LECO Chroma TOF PEGASUS HT, LECO, United States). The column (J&W Scientific, Folsom, CA, United States) used for separation was a DB-5MS capillary column coated with 5% diphenyl cross-linked with 95% dimethylpolysiloxane (30 m × 0.25 mm, 0.25 μm). Chroma TOF 4.3X software and the LECO-Fiehn Rtx5 database (LECO Corporation) were used for raw peak exacting, data baseline filtering and calibration, peak alignment, deconvolution analysis, peak identification, and integration of the peak area. The metabolomics data has been submitted to EMBL-EBI MetaboLights database with the identifier MTBLS1036.

The bioinformatic analysis of the results was performed using MetaboAnalyst^2^ 4.0 Multivariate analyses, such as principal component analysis (PCA), partial least squares discriminant analysis (PLS-DA), and orthogonal projections to latent structures-discriminant analysis (OPLS-DA), were performed. The compounds with variable importance for projection (VIP) >1.0 in the OPLS-DA and *P*-value < 0.05 were defined as significantly different metabolites between the two groups. The significantly different metabolites were further mapped into metabolic pathways using the Kyoto Encyclopedia of Genes and Genomes (KEGG) database based on pathway enrichment and topology analyses. For biomarker identification, effective peak information was imported into MetaboAnalyst 4.0. A subset of metabolites was manually selected based on each area under the curve (AUC), *P*-value and fold change (FC) as biomarkers that could be used to reflect the physiological response to SUS. The AUC value, predicted class probabilities, and cross-validation prediction were used to evaluate the effective sensitivity and specificity of selected biomarkers. The receiver operating characteristic (ROC) curve-based model evaluation (Tester) was performed using the random forests algorithm.

### Statistical Analysis

Levels of cytokines in serum, SIgA in the ileum, and metabolites in cecal contents between the two groups were analyzed with *t* test. Differences between the groups were considered statistically significant at the 5% level (*P* < 0.05). All *P*-values for the statistical tests of metabolite variations in the two groups were corrected for multiple testing using Benjamini–Hochberg false-discovery rate (FDR) method. Correlations between host immunological parameters, the intestinal microbiota and metabolite concentrations were computed with Pearson test in R using the corrplot package.

## Results

### Intestinal Metabolomic Profiles Under Simulated Weightlessness

**FIGURE 1:**
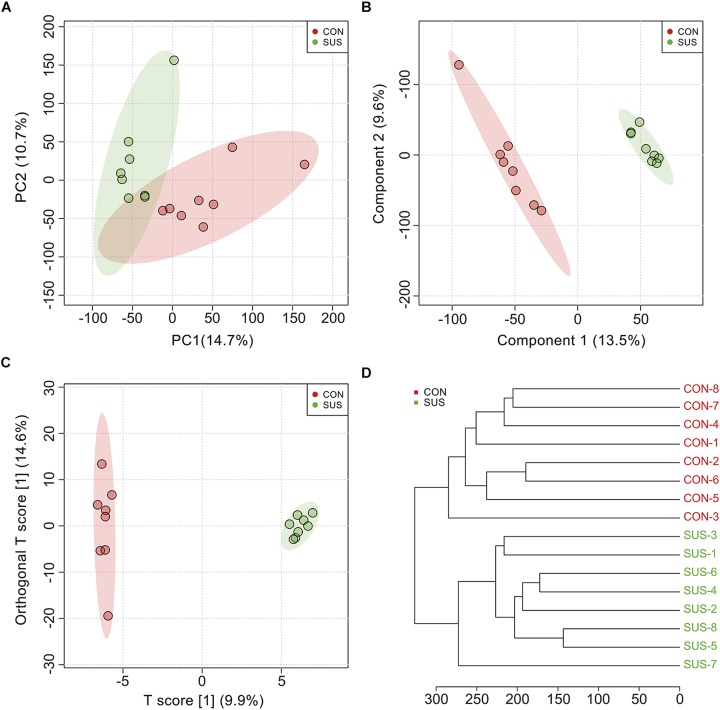
Multivariate analysis and cluster analysis of all metabolites in cecal contents from the CON and SUS groups. **(A)** The scatter plot of principal component analysis (PCA). **(B)** The scatter plot of partial least squares discriminant analysis (PLS-DA); The accuracy, goodness-of-fit (*R*^2^) and goodness-of-prediction (*Q*^2^) were 1.0, 0.995 and 0.624, respectively. **(C)** The scatter plot of orthogonal projections to latent structures-discriminant analysis (OPLS-DA); *R*^2^*X*, *R*^2^*Y*, and *Q*^2^ were 0.099, 0.875, and 0.448, respectively. **(D)** The hierarchical clustering based on the Euclidean distance and Ward clustering algorithm.

During the experiment, animals demonstrated no significant weight loss (Supplementary Figure S1). The metabolic profiles of cecal contents between the CON and SUS groups indicated that 552 effective peaks were detected in total. The PCA (Figure 1A), PLS-DA (Figure 1B) and OPLS-DA (Figure 1C) score plots showed significantly separated clusters between the two groups, with all of the samples located in the corresponding 95% Hotelling T^2^ ellipse. In addition, the clustering analysis based on the Euclidean distance and Ward clustering algorithm (Figure 1D) also demonstrated a clear distinction between the two groups, indicating the differential intestinal metabolic profiles induced by stimulated weightlessness.

**FIGURE 2:**
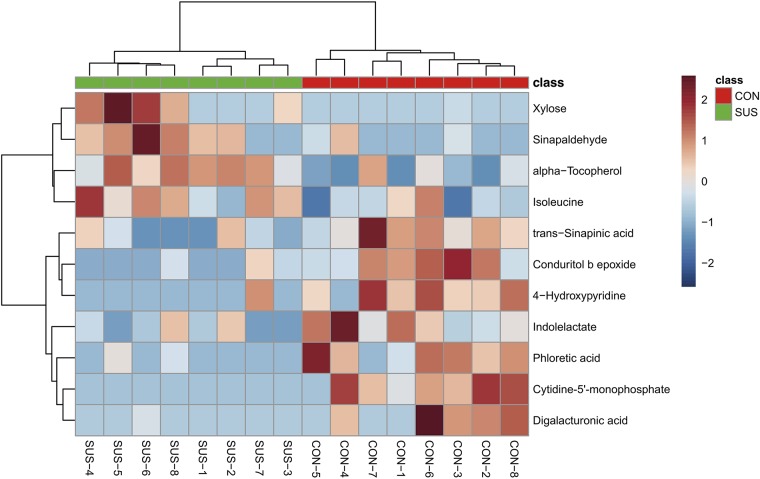
Visualization of the significantly different metabolites induced by simulated weightlessness using hierarchical cluster analysis based on the Euclidean distance and Ward clustering algorithm.

**FIGURE 3:**
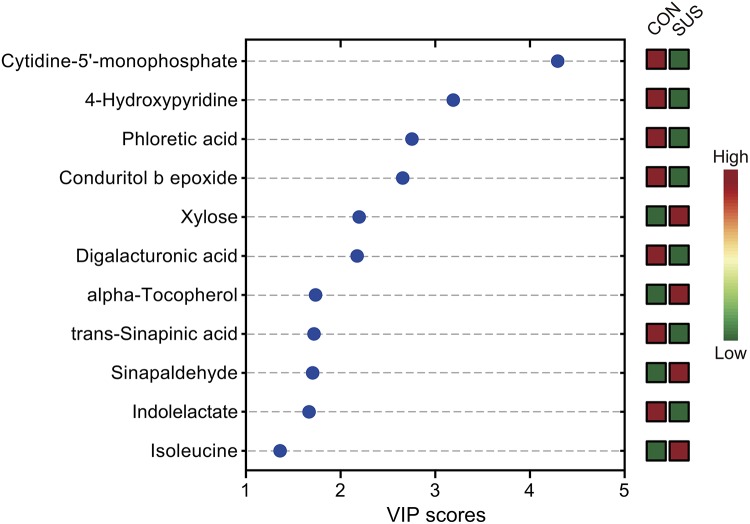
Significantly different metabolites identified by partial least squares discriminant analysis (PLS-DA). The colored boxes on the right indicate the relative concentrations of the corresponding metabolites in cecal contents from the CON and SUS groups. VIP: variable importance in projection.

After annotation, 248 metabolites were characterized and relatively quantified. As shown in Figure 2 and Supplementary Table S1, simulated weightlessness significantly enhanced the concentration of xylose, sinapaldehyde, alpha-tocopherol, and isoleucine in cecal contents, while remarkably decreased that of seven compounds, namely, *trans*-sinapinic acid, conduritol b epoxide, 4-hydroxypyridine, indolelactate, phloretic acid, cytidine-5′-monophosphate, and digalacturonic acid (*P*-value < 0.05) among them. Figure 3 shows the VIP values of the significantly different metabolites based on the PLS-DA model, which indicated that their VIP values were more than 1.

### Identification of Significantly Different Metabolic Pathways

**FIGURE 4:**
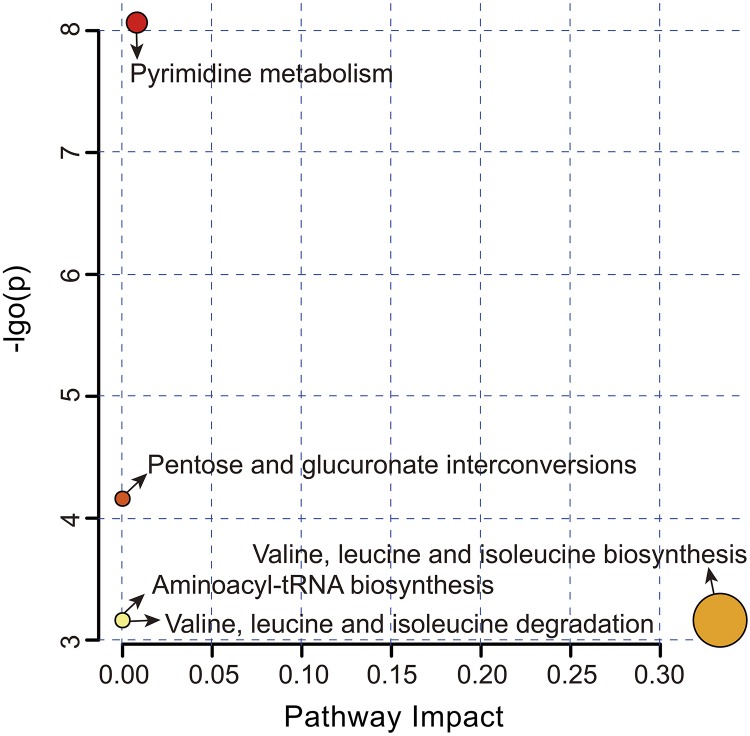
Metabolome view map of the significantly different metabolites-related metabolic pathways in the CON and SUS groups. The pathway impact in topology analysis (*x*-axis) and *P*-value in enrichment analysis (*y*-axis) are presented. The size and color of each circle represent the pathway impact value and *P*-value, respectively.

Overall, five metabolic pathways were identified after the above significantly different metabolites (SDM) were imported into KEGG (Figure 4 and Supplementary Table S2). Pyrimidine metabolism (FDR = 0.002, impact value = 0.008) and pentose and glucuronate interconversions (FDR = 0.039, impact value < 0.001) were significantly upregulated, while valine, leucine and isoleucine biosynthesis (FDR = 0.042, impact value = 0.333), valine, leucine and isoleucine degradation (FDR = 0.042, impact value < 0.001), and aminoacyl-tRNA biosynthesis (FDR = 0.042, impact value < 0.001) were remarkably downregulated in the SUS group.

### Identification of Biomarkers That Responded to Simulated Weightlessness

**FIGURE 5:**
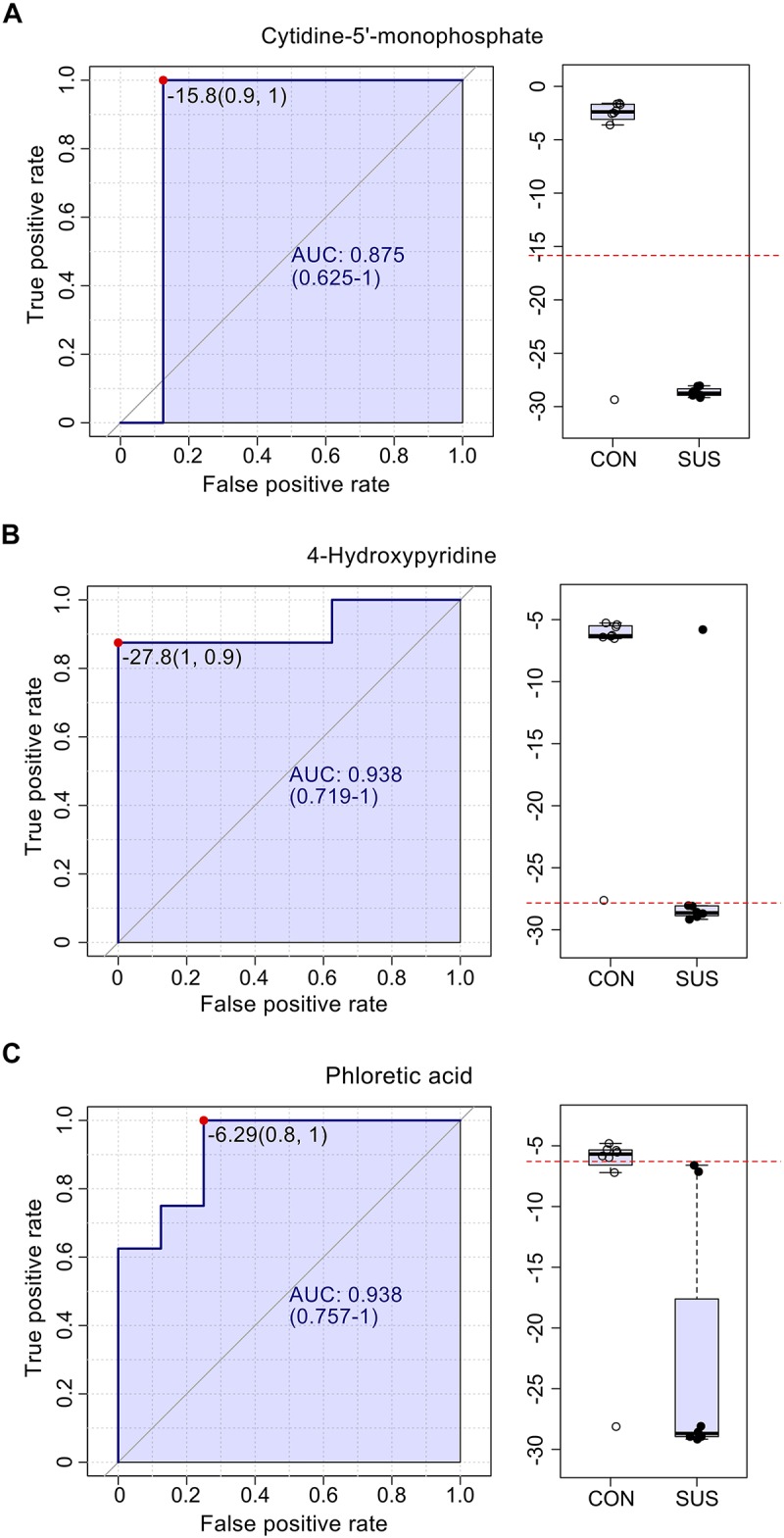
The ROC curves for cytidine-5′-monophosphate **(A)**, 4-hydroxypyridine **(B)** and phloretic acid **(C)** with AUC and respective univariate performance (box plot) in the CON and SUS groups. ROC: receiver operating characteristic; AUC: area under ROC curve.

**FIGURE 6:**
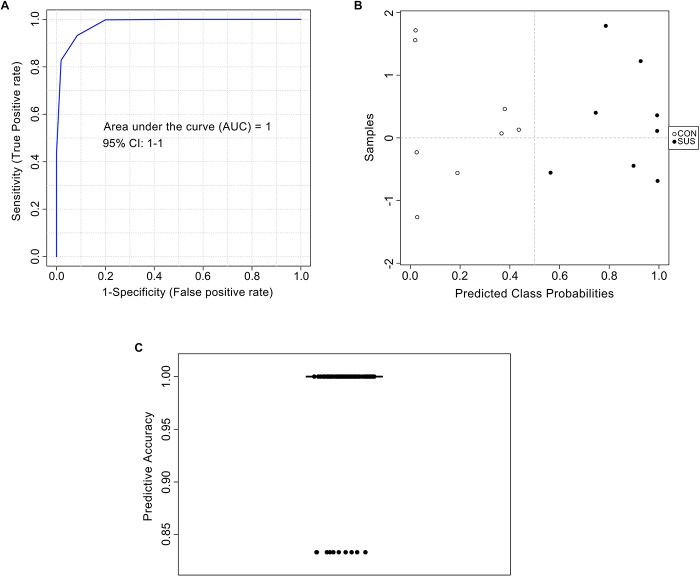
Biomarker analysis of metabolites based on the random forests algorithm. ROC view **(A)**, probability view **(B)**, and cross validation prediction **(C)** of three selected metabolites, namely cytidine-5′-monophosphate, 4-hydroxypyridine, and phloretic acid.

The AUC of cytidine-5′-monophosphate, 4-hydroxypyridine, and phloretic acid and levels of these three compounds in cecal contents are shown in Figure 5, which indicated that simulated weightlessness significantly decreased the levels of them with the AUC value of 0.875, 0.938, and 0.938. Combined with *P-*value and FC, cytidine-5′-monophosphate, 4-hydroxypyridine, and phloretic acid were manually picked as potential biomarkers in intestinal response to simulated weightlessness. The selected features were used for ROC analysis with the random forests algorithm, and the results indicated that the AUC value = 1 (Figure 6A) with an average accuracy = 0.983 based on 100 cross-validations (Figure 6C). Furthermore, the average of predicted class probabilities of each sample across the 100 cross-validations based on the balanced subsampling algorithm showed a clear cluster and separation between the CON and SUS groups (Figure 6B).

### Association of Immunological Parameters With SDM

**FIGURE 7:**
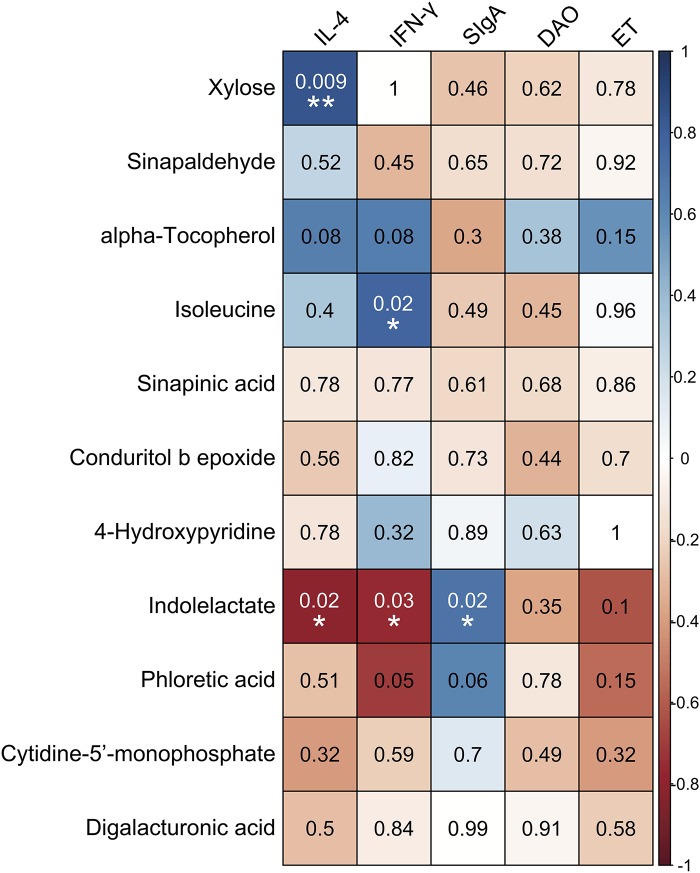
Associations among inflammation/immune indicators and the significantly different metabolites induced by simulated weightlessness by using the Pearson’s correlation coefficient with the corresponding *P*-values presented. ^∗^*P* < 0.05, ^∗∗^*P* < 0.01.

Correlations between the levels of immune-related indexes and SDM are shown in Figure 7. The results demonstrated that the concentrations of xylose, alpha-tocopherol, and isoleucine were positively correlated with the levels of IL-4 and IFN-γ in serum, while they were negatively associated with the concentration of SIgA in the ileum. In contrast, the compounds that decreased in the intestine of the SUS group, such as indolelactate, phloretic acid, and cytidine-5′-monophosphate, were negatively correlated with the levels of IL-4, IFN-γ, DAO, and ET in serum but positively associated with the concentration of SIgA in the ileum. Specifically, significantly positive correlations were observed between the concentration of xylose and the level of IL-4 (*P* = 0.009, *r* = 0.841) and those of isoleucine and IFN-γ (*P* = 0.024, *r* = 0.774) in serum. The concentration of indolelactate showed significantly negative correlations with the levels of IL-4 (*P* = 0.017, *r* = −0.8) and INF-γ (*P* = 0.025, *r* = −0.77) in serum, while there was a remarkably positive association with that of SIgA (*P* = 0.023, *r* = 0.703) in the ileum.

### Association of the Gut Microbiota With SDM

**FIGURE 8:**
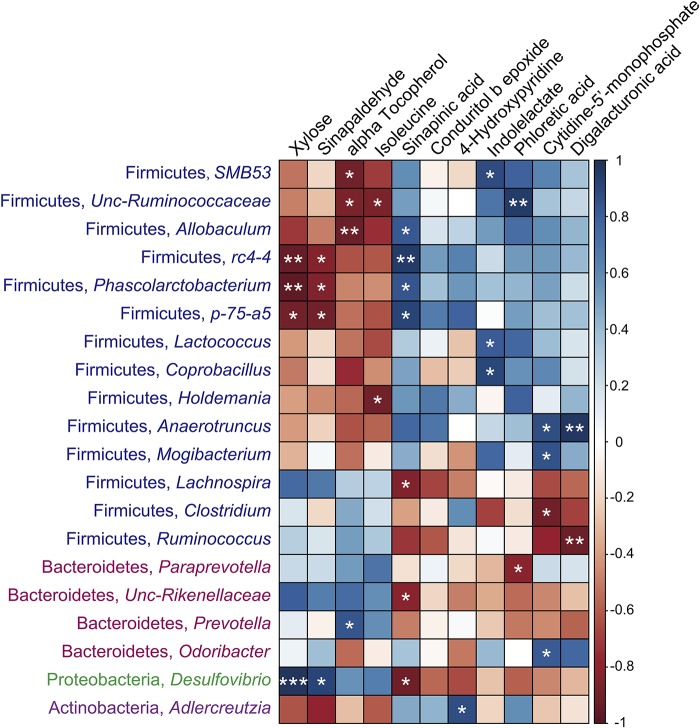
Associations among intestinal microbiomes at the genus level and the significantly different metabolites induced by simulated weightlessness by using the Pearson’s correlation coefficient. ^∗^*P* < 0.05, ^∗∗^*P* < 0.01, ^∗∗∗^*P* < 0.001.

As shown in Figure 8, the significantly different metabolites that increased in the cecal contents of the SUS group, such as xylose, sinapaldehyde, alpha tocopherol, and isoleucine, were negatively correlated with the levels of *SMB53*, *Unc-Ruminococcaceae*, *Allobaculum*, *rc4-4*, *Phascolarctobacterium*, *p-75-a5*, *Lactococcus*, *Coprobacillus*, *Holdemania*, *Anaerotruncus*, and *Mogibacterium* in Firmicutes and *Adlercreutzia* in Actinobacteria, while they were positively associated with the levels of *Lachnospira* and *Ruminococcus* in Firmicutes, *Paraprevotella* and *Unc-Rikenellaceae* in Bacteroidetes, and *Desulfovibrio* in Proteobacteria. In contrast, the compounds that decreased in the intestine of the SUS group, such as sinapinic acid, indolelactate, phloretic acid, cytidine-5′-monophosphate, and digalacturonic acid, showed remarkable opposite trends. Specifically, the concentrations of these metabolites were positively correlated with the abundance of *SMB53*, *Unc-Ruminococcaceae*, *Allobaculum*, *rc4-4*, *Phascolarctobacterium*, *p-75-a5*, *Lactococcus*, *Coprobacillus*, *Holdemania*, *Anaerotruncus*, and *Mogibacterium* in Firmicutes but negatively associated with the abundance of *Lachnospira*, *Clostridium*, and *Ruminococcus* in Firmicutes, *Unc-Rikenellaceae* and *Prevotella* in Bacteroidetes, and *Desulfovibrio* in Proteobacteria. These results suggested extensive interlinkage between the intestinal microbiota and intestinal metabolism.

## Discussion

During the spaceflight, the reduced hydrostatic pressure gradient induces redistribution of the blood from veins of the lower limbs to the head and chest, thus results in the insufficient supplies of oxygen and blood to intestine, and disturbs the normal functions of intestinal tract (Jin et al., 2018). Furthermore, the reduced splanchnic blood flow and fluid distribution decrease the intestinal motility, retard the gastrointestinal emptying, and extend the intestinal transit time thereafter (Rabot et al., 2000). These may contribute to the dysbiosis of intestinal microbiome, the disorder of intestinal metabolome and further bowel symptoms during spaceflight. However, the specific responses of intestinal metabolome under microgravity and simulated weightlessness and its relationship with the intestinal microbiome and immune characteristics remain largely unknown. The present study indicated that 21 days of tail suspension significantly induced the disturbance of intestinal metabolomic profiles. Three metabolites, cytidine-5′-monophosphate, 4-hydroxypyridine, and phloretic acid, were identified as key biomarkers that could be used to represent the intestinal metabolome and physiological differences under simulated weightlessness.

Phloretic acid is a naturally occurring polyphenol compound that might be responsible for the health-beneficial effects attributed to vegetable and fruit intake based on metabolism by intestinal bacteria. It has been demonstrated that phloretic acid from the colonic microbiota exerted anti-inflammatory protection on colon fibroblasts (Larrosa et al., 2009; van Duynhoven et al., 2013). As one of the important microbial tryptophan catabolites, indolelactate is suggested to be able to activate the immune system, enhance intestinal epithelial barrier function, stimulate gastrointestinal motility, and exert anti-inflammatory and antioxidative effects as well as modulate the gut microbial composition (Roager and Licht, 2018). The present study demonstrated that simulated weightlessness significantly decreased levels of phloretic acid and indolelactate in cecal contents. Furthermore, these two metabolites showed a negative association with the concentration of IL-4 and IFN-γ and a positive correlation with that of SIgA in the ileum. Downregulation of phloretic acid and indolelactate might be a trigger of inflammation through the activation of the TLR4/MyD88/NF-κB signaling pathway under simulated weightlessness, the results of which were reported in our previous study (Jin et al., 2018). A recent study reported that a 340-day space mission onboard the International Space Station decreased the abundance of several small-molecule markers of gut microbial metabolism with anti-inflammatory effects, such as 3-indole propionic acid (Garrett-Bakelman et al., 2019). These results are in agreement with our findings.

The inherent environment during spaceflight, such as microgravity, cosmic radiation, and hypomagnetic field, could induce injury or physical and physiological stress and cause a cumulative impact on the body (Garrett-Bakelman et al., 2019). Growing investigations have clearly demonstrated that microgravity may affect the oxidative stress response not only in hosts but also in a variety of bacteria (Jayroe et al., 2012; Aunins et al., 2018; Pavlakou et al., 2018). The related oxidative stress is involved in the progression of physiological alterations, such as immune dysfunction, inflammation, muscle atrophy, bone loss, and metabolism disorder (Lawler et al., 2003; Jackson, 2009; Wauquier et al., 2009; Bergouignan et al., 2016). Furthermore, microgravity could improve antibiotic-resistance traits of bacteria due to the oxidative stress response, thus increasing bacterial virulence and creating a threat to spaceflight missions (Aunins et al., 2018). Sinapinic acid was found to have significant protective potential against oxidative stress-induced diseases and aging (Nićiforović and Abramovič, 2014; Chen, 2016). In addition, 4-hydroxypyridine, substance derived from the ergoline structure, may exhibited antioxidant activities through free radicals binding and formation inhibition (Štětinová and Grossmann, 2000). In the present study, it was found that simulated weightlessness remarkably decreased the amount of sinapinic acid, 4-hydroxypyridine and indolelactate, which suggested the high oxidative status and oxidative stress within the intestinal tract that might contribute to the inflammation and breakdowns of intestinal homeostasis. Some literature has reported oxidative bursts under space environments and simulated microgravity or weightlessness conditions (Yamauchi et al., 2002; Jayroe et al., 2012; Seawright et al., 2017; Pavlakou et al., 2018). Mechanisms of interactions between intestinal microbiota metabolites and oxidative stress-induced intestinal dysfunction under microgravity need to be further investigated.

To reveal the systematic effect of the significantly different metabolites under simulated weightlessness, they were imported into KEGG to characterize the most influential pathways. Pentose and glucuronate interconversions and valine, leucine and isoleucine metabolism pathways were confirmed to contribute to significantly upregulated xylose and isoleucine in cecal content. In our previous study, we reported that the intestinal microbiome under simulated weightlessness might have a depressed capacity for energy harvesting due to the slowdown of intestinal peristalsis, the extension of gut transit time and the resulting reduced nutrients provided to the gut microbiota (Jin et al., 2018). As one of the carbohydrate metabolism subcategories, the improvement of the pentose and glucuronate interconversions pathway is coincident with the fact that the gut microbiome needs to metabolize more carbohydrates to resist more complex environments, such as the simulated weightlessness used in the current research (Zhou et al., 2014). These results are also in agreement with the decreased level of conduritol B epoxide (a β-glucosidase inhibitor) (Mercer et al., 2013), which might also contribute to the improvement of carbohydrate metabolism in the SUS group.

Isoleucine is a branched-chain amino acid involved in the valine, leucine and isoleucine metabolism pathways. The branched-chain amino acids can be metabolized by Stickland reactions and produce branched-chain fatty acids, which typically serve as electron donors (Macfarlane and Macfarlane, 1997). Abnormally increased branched-chain amino acid concentrations are good biomarkers for early detection of metabolic diseases (Zhang et al., 2017). Meanwhile, the decreased levels of isoleucine and cytidine-5′-monophosphate in the SUS group are associated with aminoacyl-tRNA biosynthesis and pyrimidine metabolism, which may positively correlate with intestinal inflammation and disruption (Bagdas et al., 2011; Yao and Fox, 2013). These findings are consistent with our previous report that simulated weightlessness significantly downregulates the genetic information processing pathway (Jin et al., 2018).

Recently, microgravity or simulated weightlessness has been proven to be a crucial modulator of the intestinal microbiota (Li et al., 2015; Ritchie et al., 2015; Shi et al., 2017; Garrett-Bakelman et al., 2019). For instance, long-duration space missions and hindlimb unloading influenced the intestinal microbiota, with changes in the Firmicutes to Bacteroidetes ratio (F/B ratio) (Jin et al., 2018; Garrett-Bakelman et al., 2019). The altered metabolites produced by the dysbiotic microbiome serve as intermediaries between not only the microbiota and host but also the microbiome inside the intestinal ecosystem. Microbial metabolites further regulate the host physiology that contributes to the health status of the host. In this study, we examined the association between metabolomic data and microbiome signatures. It was found that xylose, sinapaldehyde, alpha tocopherol, and isoleucine, which were upregulated by SUS, were negatively correlated with the levels of genera such as *SMB53*, *Allobaculum*, *rc4-4*, *Lactococcus*, *Holdemania*, *Anaerotruncus*, and *Mogibacterium* in Firmicutes, while they were positively associated with the levels of some genera such as *Paraprevotella* and *Unc-Rikenellaceae* in Bacteroidetes and *Desulfovibrio* in Proteobacteria. Interestingly, the metabolites that were downregulated by SUS, such as sinapinic acid, indolelactate, phloretic acid, cytidine-5′-monophosphate, and digalacturonic acid, showed distinct opposite trends. The physiological significance of such dysbiosis was discussed in our previous study (Jin et al., 2018). Supplementation of sinapinic acid could obviously improve the level of butyrate-producing bacteria in the *Lachnospiraceae* family, suppress the growth of species associated with inflammation and diseases, such as *Bacteroides* and *Desulfovibrionaceae* spp., thus alleviate oxidative stress in high-fat diet-fed rats (Yang et al., 2019). The present study suggested that metabolites such as sinapinic acid, indolelactate, phloretic acid, and digalacturonic acid are negatively associated with the genera in Bacteroidetes and *Desulfovibrio*, and the low levels of these compounds may induce inflammation in the intestinal tract. Furthermore, xylose could be fermented by *Bacteroides* spp. and *Prevotella* spp. to produce short-chain fatty acids (Obregon-Tito et al., 2015). The high level of xylose in the intestinal tract implies that less xylose was used by the above microbiome as a carbohydrate metabolizer, which may result in low levels of anti-inflammatory and health-beneficial short-chain fatty acids. We did not detect short-chain fatty acids in cecal contents due to volatilization during the sample pretreatment for GC-TOF/MS (Jin et al., 2019). The levels of short-chain fatty acids in the intestine under simulated weightless need to be demonstrated in further study. In addition, digalacturonic acid was found to be able to prevent adhesion of *Escherichia coli* O157:H7 to human colonic cells (Olano-Martin et al., 2003); the decreased antiadhesive property induced by downregulation of digalacturonic acid under simulated weightlessness may contribute to the dysbiosis of the gut microbiota.

Finally, we analyzed the association of significantly different metabolites with intestinal immune function under simulated weightlessness. In our previous research, we showed an increase in proinflammatory cytokines, a decrease in SIgA, and activation of the TLR4/MyD88/NF-κB signaling pathway under simulated weightlessness (Jin et al., 2018). The present study demonstrated that the levels of downregulated compounds such as indolelactate, phloretic acid, cytidine-5′-monophosphate, and digalacturonic acid in the intestinal tract were positively correlated with SIgA in the ileum but negatively associated with the levels of IL-4, IFN-γ, DAO, and ET in serum. Furthermore, four significantly different metabolites upregulated by SUS showed a distinct opposite trend. Specifically, indolelactate showed significant connections not only with the concentrations of SIgA, IL-4, and IFN-γ but also with the abundance of *Lactococcus* and *Coprobacillus.* The probiotic activities of *Lactococcus* and *Coprobacillus* have been extensively suggested (Ye et al., 2018); meanwhile, indolelactate is involved in the regulation of the immune system due to its antiinflammatory bioactivity, as we mentioned above (Roager and Licht, 2018). These results imply that intestinal metabolites could be considered intermediaries between the microbiota and immune function.

In summary, the present research demonstrated the disturbance of the intestinal metabolomic profile induced by simulated weightlessness. Pyrimidine metabolism, pentose and glucuronate interconversions and valine, leucine and isoleucine metabolism were identified as the main metabolic pathways contributing to the significantly different metabolites. Cytidine-5′-monophosphate, 4-hydroxypyridine, and phloretic acid were characterized as key biomarkers that responded to simulated weightlessness. Furthermore, the disruption of intestinal metabolomes was correlated with immune dysfunction and intestinal dysbiosis. These metabolic characteristics provide crucial candidates for therapeutic targets for metabolic disorders under microgravity. Prebiotic and probiotic supplementation based on sufficiently different metabolites and microbiomes selected in this study might be explored as an efficient nutritional countermeasure to avoid unbalanced intestinal homeostasis of crewmembers.

## Data Availability Statement

All datasets generated for this study are included in the manuscript/Supplementary Files.

## Ethics Statement

The animal study was reviewed and approved by Institutional Animal Care and Use Committee of Northwestern Polytechnical University.

## Author Contributions

MJ conceived the project and designed the study. KZ provided content knowledge and additional suggestions for the design of the study. JW, HaZ, and HoZ performed the experiments. MJ and KZ analyzed the data, created figures, and drafted the manuscript. All authors reviewed the manuscript.

## Conflict of Interest

HoZ was employed by Dalian Chengsan Animal Husbandry Co., Ltd. The remaining authors declare that the research was conducted in the absence of any commercial or financial relationships that could be construed as a potential conflict of interest.

## Footnotes

1 https://huttenhower.sph.harvard.edu/galaxy/

2 http://www.metaboanalyst.ca

## Supplementary Material

FIGURE S1 The body weight of rats from the CON and SUS groups after 21 days of experiment.

TABLE S1 Identification of significantly different metabolites in cecal contents between CON and SUS groups.

TABLE S2 Metabolomic pathway analyses of cecal contents between CON and SUS groups.
