# Supplementary material for: Simulated Weightlessness Perturbs the Intestinal Metabolomic Profile of Rats
Source: Front Physiol. 2019 Oct 15;10:1279. doi: 10.3389/fphys.2019.01279 (PMC6803529; doi:10.3389/fphys.2019.01279)
Supplement: TABLE S1 — Identification of significantly different metabolites in cecal contents between CON and SUS groups. [file Table_1.docx]

**Supplementary Table S1.** Identification of significantly different metabolites in cecal contents between CON and SUS groups

| Metabolite name | RT^a^ | Mass | Similarity | Relative abundance | | log2(FC)^b^ | *t*-test | | VIP | AUC |
| --- | --- | --- | --- | --- | --- | --- | --- | --- | --- | --- |
|  |  |  |  | CON | SUS |  | *P* value | FDR |  |  |
| Cytidine-5'-monophosphate | 22.905 | 168 | 339 | 0.199±0.119 | 0.000±0.000 | 26.275 | <0.001 | 0.087 | 4.294 | 0.875 |
| 4-Hydroxypyridine | 8.970 | 152 | 713 | 0.015±0.008 | 0.002±0.006 | 2.7242 | 0.004 | 0.189 | 3.190 | 0.938 |
| Phloretic acid | 16.605 | 179 | 726 | 0.018±0.011 | 0.002±0.004 | 3.0741 | 0.002 | 0.140 | 2.756 | 0.938 |
| Conduritol b epoxide | 18.462 | 239 | 325 | 0.016±0.008 | 0.003±0.005 | 2.3527 | 0.002 | 0.154 | 2.656 | 0.938 |
| Xylose | 15.255 | 307 | 899 | 0.000±0.001 | 0.012±0.012 | -5.3035 | 0.016 | 0.321 | 2.197 | 0.844 |
| Digalacturonic acid | 26.195 | 397 | 493 | 0.013±0.013 | 0.001±0.002 | 4.5261 | 0.014 | 0.321 | 2.177 | 0.719 |
| alpha-Tocopherol | 28.118 | 237 | 713 | 0.006±0.007 | 0.017±0.005 | -1.6036 | 0.002 | 0.140 | 1.737 | 0.938 |
| trans-Sinapinic acid | 21.369 | 268 | 242 | 0.020±0.009 | 0.008±0.008 | 1.4339 | 0.009 | 0.295 | 1.720 | 0.875 |
| Sinapaldehyde | 20.0578 | 174 | 353 | 0.005±0.007 | 0.020±0.015 | -2.1319 | 0.021 | 0.364 | 1.706 | 0.797 |
| Indolelactate | 20.667 | 290 | 330 | 0.017±0.010 | 0.006±0.015 | 1.4244 | 0.026 | 0.396 | 1.667 | 0.844 |
| Isoleucine | 10.811 | 158 | 948 | 0.214±0.166 | 0.393±0.015 | -0.8747 | 0.042 | 0.508 | 1.362 | 0.797 |

^a^RT=retention time. ^b^FC=fold change, mean value of peak area obtained from the CON group/mean value of peak area obtained from the SUS group.
