# Supplementary material for: Simulated Weightlessness Perturbs the Intestinal Metabolomic Profile of Rats
Source: Front Physiol. 2019 Oct 15;10:1279. doi: 10.3389/fphys.2019.01279 (PMC6803529; doi:10.3389/fphys.2019.01279)
Supplement: TABLE S2 — Metabolomic pathway analyses of cecal contents between CON and SUS groups. [file Table_2.docx]

**Supplementary Table S2.** Metabolomic pathway analyses of cecal contents between CON and SUS groups

| Pathway Name | Hit^a^ | Enrichment Analysis | | Topology Analysis  Impact Value |
| --- | --- | --- | --- | --- |
|  |  | *P* Value | Holm *P*^b^ |  |
| Pyrimidine metabolism | 1/41 | < 0.001 | 0.002 | 0.008 |
| Pentose and glucuronate interconversions | 1/14 | 0.016 | 0.062 | < 0.001 |
| Valine, leucine and isoleucine biosynthesis | 1/11 | 0.042 | 0.127 | 0.333 |
| Valine, leucine and isoleucine degradation | 1/38 | 0.042 | 0.127 | < 0.001 |
| Aminoacyl-tRNA biosynthesis | 1/67 | 0.042 | 0.127 | < 0.001 |

a: The number of metabolites that matched in the KEGG pathway. b: The statistical *P* values that were further adjusted using the Holm-Bonferroni method.
